# First record of natural infection by *Angiostrongylus cantonensis* (Nematoda: Metastrongyloidea) in *Tanychlamys indica* (Godwin-Austen, 1883) in the city of São Paulo, Brazil

**DOI:** 10.1590/0074-02760240192

**Published:** 2025-03-31

**Authors:** Dan Jessé Gonçalves da Mota, Sylvio Cesar Rocco, Liliane Ré Di Luca, Jailson Apóstolo dos Santos, Eliana Fernandes Pavani Werneck, Amanda de Oliveira Baccin, Ricardo Gava, Vera Lucia Pereira-Chioccola, Leyva Cecília Vieira de Melo

**Affiliations:** 1 Secretaria Municipal da Saúde de São Paulo, Divisão de Vigilância de Zoonoses, Laboratório de Pesquisa e Identificação de Fauna Sinantrópica, São Paulo, SP, Brasil; 2 Secretaria Municipal da Saúde de São Paulo, Vigilância Ambiental, Unidade de Vigilância em Saúde, São Paulo, SP, Brasil; 3 Instituto Adolfo Lutz, Centro de Parasitologia e Micologia, Núcleo de Enteroparasitas, São Paulo, SP, Brasil; 4 Instituto Adolfo Lutz, Centro de Parasitologia e Micologia, Laboratório de Biologia Molecular de Parasitas e Fungos, São Paulo, SP, Brasil

**Keywords:** Angiostrongylus cantonensis, Macrochlamys indica, exotic species, eosinophilic meningitis, neuroangiostrongyliasis

## Abstract

**BACKGROUND:**

*Tanychlamys indica* (Godwin-Austen, 1883) was reported as a serious pest in India. The snails are voracious and feed on a wide range of commercial crops. It has also been identified as an intermediate nematode host of *Angiostrongylus cantonensis* in Bombay, India. *T. indica* was recently introduced in Brazil by international trade of citrus fruit seedlings. First in the State of Santa Catarina and then in Paraná. Recently, it has been detected in the city of São Paulo threatening to spread to other Brazilian states.

**OBJECTIVES:**

We report the first record, in Brazil, of the natural infection by L3 larvae of *A. cantonensis* isolated from *T. indica* collected in the Vila Leopoldina neighbourhood, located in the west zone of São Paulo city.

**METHODS:**

In January 2023, a team from LABFAUNA and UVIS Lapa collected 36 molluscs identified as *T. indica* in Vila Leopoldina, São Paulo city. Of these, 20 molluscs were subjected to individual parasitological analysis at the Instituto Adolfo Lutz, using the modified Rugai methodology.

**FINDINGS:**

A total of 145 larvae were identified morphologically and classified according to Ash’s criteria. These larvae were identified as third - stage larvae (L3) of *A. cantonensis* by real time polymerase chain reaction (PCR).

**MAIN CONCLUSIONS:**

It is evident that further research is imperative to map the distribution of *T. indica* in Brazil and to assess its potential as an intermediate host for the nematode *A. cantonensis*, as well as the economic risks to agriculture. Over the past two decades, human cases of neuroangiostrongyliasis have been documented in the Southeast, North, Northeast, and South regions of Brazil. Additionally, there are records of natural infection with *A. cantonensis* in molluscs and rodents.

Terrestrial gastropods are agricultural pest species with significant importance in the world. They cause significant economic damage in a range of agricultural crops, including vegetables, fruit trees, field crops, ornamentals and medicinal plants.^1^ ^,^ ^2^

The human activities caused the dispersion of the numerous species of terrestrial molluscs across a vast geographical area. As a consequence, they adapted to new habitats, where they are now regarded as exotic species.^3^ ^,^ ^4^

Synanthropic gastropods are species capable of long-distance dispersal. Thus, they had become invasive in urban and agricultural environments around the world.^5^

Due to their abundance and biomass, terrestrial gastropods are capable of depositing considerable amounts of mucus and faecal material on crops. This process results in reduction of crop value and quality. In addition, terrestrial gastropods cause wounds in plants, facilitating the entry of pathogenic microbes.^6^ ^,^ ^7^ Furthermore, various terrestrial gastropods act as intermediate hosts for parasites and pathogens that pose risks to animal and human health.^8^

*Angiostrongylus cantonensis* (Chen, 1935) is a parasite that affects the pulmonary arteries and right ventricle of rats and was described in Canton in China. Since then, they have been identified in various locations as the Pacific Islands, Asia, Australia, Africa, and some Caribbean islands. Recently, it has been detected in the Americas.^9^ The definitive hosts for *A. cantonensis* are *Rattus norvegicus* (Berkenhout, 1769) and *Rattus rattus* (Linnaeus, 1758).^10^

Accidental infection in humans by *A. cantonensis* occurs when they consume raw or undercooked snails, slugs, and paratenic hosts (*e.g.*, shrimps, crabs, lizards, frogs, land planarians, and centipedes) or vegetables contaminated with mucus from infected molluscs.^9^ ^,^ ^11^ ^,^ ^12^ The increase of larvae release occurs when these are exposed to stressful situations.^13^ In contrast to the natural definitive host, *A. cantonensis* does not complete its life cycle in humans. Instead, the larvae die when they reach in the brain, causing an intense inflammatory reaction that result in neuroangiostrongyliasis.^14^ ^,^ ^15^

The territorial molluscs, as intermediate hosts are responsible for transmission of angiostrongyliasis to humans. *A. cantonensis* has already been identified in all Brazilian regions as in Southeast,^16^ ^,^ ^17^ ^,^ ^18^ ^,^ ^19^ Northeast,^20^ ^,^ ^21^ ^,^ ^22^ North^23^ ^,^ ^24^ ^,^ ^25^ and South.^11^ ^,^ ^15^ ^,^ ^16^ Therefore, distant regions from one another can exhibit comparable terrestrial mollusc faunas comprising species of exotic nature.^5^ In Brazil, as in other countries, the increase of human activities has resulted in the rapid introduction of mollusc species. Thereby, an in-depth study of their fauna is necessary.^3^ *Tanychlamys* (Benson, 1834) is a genus that comprises more than 100 species that are widely distributed in India and Southeast Asia.^26^ ^,^ ^27^ *Tanychlamys indica* (Godwin-Austen, 1883) is one most common species in India.^26^ ^,^ ^28^ Previously this species was known as *Macrochlamys indica* (Godwin-Austen, 1883).^29^ The horntail snail, *T. indica*, is a voracious herbivore with the capacity to completely devour young seedlings. It is a pervasive pest in vegetable and ornamental plant nurseries in India and Bangladesh with potential to become invasive.^30^ ^,^ ^31^ ^,^ ^32^

In the USA, *T. indica* is included on the list of quarantine plant pests, due to the potential threat it poses to agricultural interests.^33^ Singh et al.^30^ documented the presence of *T. indica* on citrus and guava seedlings in Punjab, India. The snails exhibit a high degree of trophic specialisation, feeding on a diverse array of commercially cultivated plants, including cabbage, beans, lettuce, moringa, yams, chrysanthemums, and cucurbits.^34^ ^,^ ^35^ ^,^ ^36^ The *T. indica* introduction in Brazil is a recent phenomenon, potentially linked to the trade in citrus fruit seedlings in the State of Santa Catarina and, subsequently, in Paraná.^37^ ^,^ ^38^

On 29 November 2022, the Vigilância Ambiental da Unidade de Vigilância em Saúde Lapa (UVIS Lapa) conducted surveillance and control actions on synanthropic animals. The results produced a documentation of *T. indica* specimen in a small wood/garden located in a municipal school in Vila Leopoldina neighbourhood, in the western zone of the city São Paulo. Subsequently, on 19 January 2023, an investigation joint with the Laboratório de Identificação e Pesquisa de Fauna Sinantrópica (Labfauna) DVZ and UVIS Lapa resulted in a capture of 36 specimens of *T. indica*, comprising juveniles and adults.

This report constitutes the first description of *T. indica* naturally infected by *A. cantonensis*, L3 larvae in Brazil. The infections were identified in four molluscs. The specimens were analysed in Instituto Adolfo Lutz.

## MATERIALS AND METHODS

**Fig. 1:**
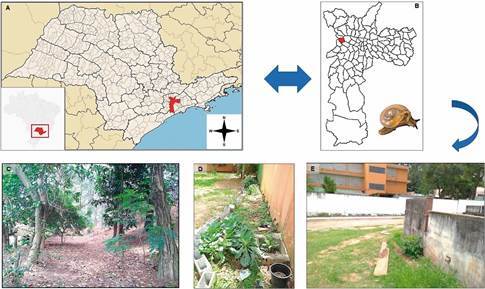
(A-B) map of São Paulo State, with São Paulo city and the administrative district of Vila Leopoldina highlighted in red. The blue arrow indicates a general view of the *Tanychlamys indica* collection areas in the Vila Leopoldina neighbourhood in January 2023. (C-D) woods and school garden. (E) a general view of the collection point near a grave in the Cemitério da Lapa- São Paulo/SP.

*Study areas* - The malacological research was conducted at a municipal school situated on the west side of the municipality of São Paulo, SP, with geographical coordinates 23°32’9.708” S, 46°43’27.743”. The site encompasses an area of 7.557.36 m^2^. The educational establishment is a primary and elementary school, comprising grades 1 to 9, with a capacity of 755 places for children and young people aged between seven and 15 years. The outdoor area of the school includes a vegetable garden, a playground, a sports court, a garden, and a green area (2.897.07 m²), which was the site of the active searches for molluscs. The mollusc collection was extended to encompass a garden area and the graves of the Cemitério da Lapa, due to its proximity to the wall of the school under investigation (Fig. 1).

*Molluscs collected and Angiotrongylid identification* - In November 2022 occurred by UVIS Lapa, the first record of a land snail specimen collected in the educational establishment. Subsequently, on 19 January 2023 a grouped action was undertaken by LABFAUNA/DVZ and UVIS Lapa, whereby the school’s garden, flowerbeds, vegetable garden, sports court, playground, and woods, as well as a garden area near the cemetery graves, were inspected.

The investigation was conducted during the day in locations where gastropods could be found, including toys in the playground, vegetable garden beds, soil under leaves, branches, plants, stones, mud bricks, cement blocks, roof tiles, pieces of wood, rubble, cracks and crevices in the masonry of plant beds and walls, shaded environments under tree canopies, and recesses in the masonry wall and graves. The 36 gastropods were placed in plastic containers lined with moist paper towels for subsequent analysis. The containers were stored in a polystyrene box to ensure the maintenance of optimal humidity levels. Next, the snails were conveyed to LABFAUNA, where they were accommodated in a terrarium and nourished with lettuce leaves that had been sanitised in a 1% sodium hypochlorite solution. Taxonomic identification was based on an analysis of the snail’s shell and body morphology.^37^ ^,^ ^39^ Shell diameter measurements were obtained utilising a Digimess Stainless Hardened digital calliper, with a precision of 0.05mm/1/128 inch.

In February 2023, 20 specimens of terrestrial gastropods were collected and sent to Instituto Adolfo Lutz for nematode larval extraction using the modified Rugai methodology.^40^ A total of 20 molluscs were individually digested as previously described by Mota et al.^19^ The L3 larvae, which were recovered alive, were morphologically identified and classified based on Ash’s criteria.^41^ Next, the specimens were transferred to an excavated plate utilising a Carl Zeiss stereomicroscope (Stemi 500). Concurrently, 30 larvae were fixed in 70% alcohol and photographed using a digital camera attached to a standard optical microscope (Jenaval). The images were analysed using the AxioVision 4.8 software. Furthermore, a group of 20 L3 larvae recovered from natural mollusc infections was frozen at -20ºC for molecular analysis.

*Angiostrongylus species: molecular identification* - The L3 larvae samples were molecularly analysed to identify *Angiostrongylus* species. Prior to DNA extraction, the larvae were placed in 1.5 mL tubes, crushed, and digested in a lysis buffer (Tris-HCl, 10 mM, pH 8.0; EDTA, 10 mM; SDS, 0.5%; N-laurylsarcosyl, 0.01%; proteinase K, 100 μg/mL). The larvae were disrupted by shaking with glass beads in a single cycle of 50 oscillations per second for five minutes with the aid of the TissueLyser LT apparatus (Qiagen). The mixtures were incubated at 56ºC for 20 min, or until complete tissue lysis. Next, DNA samples were extracted using the QIAamp DNA Mini Kit (Qiagen), in accordance with the manufacturer’s instructions. The DNA concentrations and purity were determined by measuring the ratio of the optical density (OD) at 260 and 280 nanometres (nm) in a NanoDrop ONE spectrophotometer (Thermo Scientific).

DNA molecules were submitted to conventional polymerase chain reaction (cPCR) using the molecular markers NC1 (5’ ACGTCTGGTTCAGGGTTGTT 3’) and NC2 (5’ TTAGTTTCTTTTCCTCCGCT 3’), which amplify a specific region of the internal transcribed spacer 2 (ITS2) of the rDNA.^42^ ^,^ ^43^ The amplifications were performed using a mixture containing 25 pmol of each molecular marker, 12.5 μL of Go Taq Green Master Mix (Promega), which comprises 1 unit of Taq DNA polymerase, 10 mM Tris-HCl (pH 8.5), 50 mM KCl, 1.5 mM MgCl_2_, and 200 mM of each dNTP. In each amplification reaction were included a negative control (ultrapure water) and two positive DNA controls: from *A. cantonensis,* Hawaiian strain and *A. costaricensis*, Crissiumal/RS strain. The thermal cycles were conducted in a Veriti®-96 Well Thermal Cycler/Applied Biosystems, with the following conditions: an initial denaturation at 94ºC for 5 min; 30 cycles of denaturation at 94ºC for 60 s, annealing at 58ºC for 60 s, and extension at 72ºC for 60 s; and a final extension at 72ºC for 10 min.

*Angiostrongylus cantonensis* was further identified by real-time PCR (qPCR) using the molecular marker AcantITS1, which amplifies a region of the ITS1 of *A. cantonensis*.^44^ The sequence designers were: forward (5′ TTCATGGATGGCGAACTGATAG 3′); reverse (5′ GCGCCCATTGAACATTATACTT 3′) and probe (5′ ATCGCATATCTACTATACGCATGTGACACCTG 3′) labelled with FAM (6-carboxyfluorescein) and BHQ1 (Black Hole Quencher 1) at the 5′ and 3′ ends, respectively. The reactions were performed in a final volume of 20 µL, comprising DNA samples (3 µL of DNA up to 100 ng/µL), and 10 µL of 2 X TaqMan Universal PCR Master Mix, 18 µM of molecular markers and 5 µM of the probe. Amplifications were conducted on an Agilent AriaMax Real-Time PCR system, employing a thermal profile comprising two minutes at 50ºC and 10 min at 95ºC. Subsequently, 40 cycles were conducted at 95ºC for 15 s and 60ºC for 1 min. The results were automatically determined by the equipment and expressed as a cycle threshold value (CT), which indicated the quantity of the target gene at which the fluorescence exceeded a present threshold.

## RESULTS

**Fig. 2:**
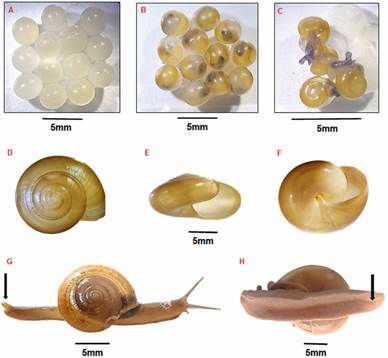
(A) whitish eggs of *Tanychlamys indica* in the first days of laying. (B) light brown eggs days after laying. (C) newly hatched specimens. (D) shell of *T. indica* in apical view. (E) shell opening. (F) basal view. (G) live specimen of *T. indica* collected in Vila Leopoldina neighbourhood arrow indicates caudal horn. (H) preserved specimen of *T. indica* collected in Vila Leopoldina neighbourhood. The arrows show in details the tripartite sole.

The 36 molluscan specimens collected in the school garden and areas in Cemitério da Lapa exhibited a depressed, fragile, smooth, thin, translucent, and perforated shell. Their surface displayed fine growth lines, a light brown periostrum, a demarcated suture, and a slightly elevated spiracle. The aperture is dextral, semilunar and oblique, with a thin peristome, a curved columellar lip (which is oblique, vertical and slightly reflexed, partially covering the umbilicus), and an outer lip that is sharp and turns varied from five to six (Fig. 2D-F). The shell diameter ranged from 7.4 mm to 21 mm. The body was characterised by a reticulated, greyish epidermis, which became darker in the cephalic region and exhibited more intense pigmentation along the tentacles, while the colouration was lighter near the sole of the foot. In response to disturbance, the animal released a yellowish-green mucus. The sole was tripartite, while the posterior dorsal end of the foot was longitudinally bipartite, forming a raised protuberance known as the caudal horn (Fig. 2G-H). This is a characteristic feature of the species *T. indica*.

In February 2023, 16 specimens of *T. indica* were formally catalogued in the malacological collection of the LABFAUNA, bearing the designation 40215-1/2023. At the LABFAUNA collection, one of the molluscan species with a shell diameter of 18.4 mm was observed to lay a clutch of 53 eggs. The eggs were observed to be round, translucent, and whitish in colour. It was observed that the eggs underwent a change in colour, becoming light brown as the embryos developed. The hatching process occurred between 18 and 21 days after the initial deposition of the eggs (Fig. 2A-C).

**Fig. 3:**
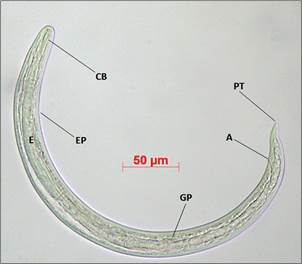
light microscopy of a third-stage larvae (L3) of *Angiostrongylus cantonensis* isolated from *Tanychlamys indica* collected at a municipal school in the Vila Leopoldina neighbourhood in February 2023. At the anterior end of the L3 larvae (CB) buccal capsule, (EP) excretory pore, (E) oesophagus. Posterior end of larva showing pointed tail (PT), genital primordium (GP) and anus (A).

A total of 145 L3 larvae were obtained from a batch of 20 *T. indica* specimens that were individually digested, with an infection rate of 20% (n = 4). In the gastropods that exhibited natural infection by Angiostrongylid larvae, the shell diameter ranged from 19 to 21 mm, with the number of larvae recovered varying from two to 92. Specifically, two, 21, 30, and 92 larvae were isolated from four specimens of *T. indica*. The 30 L3 larvae employed for the morphometric study exhibited a filiform body, furrowed cuticle, rounded anterior end, elongated buccal capsule, and a claviform bulb situated at the terminus of a lengthy oesophagus. The excretory pore was identified in the mid-region of the oesophagus. Furthermore, a genital primordium was observed in the posterior third of the intestine, and the larvae exhibited a pointed tail (Fig. 3). The results of the morphometric analyses of the 30 specimens are presented in Table I.

**TABLE I:** Morphometry of L3 larvae (N = 30) isolated from *Tanychlamys indica* naturally infected with *Angiostrongylus cantonensis*

Measures	Mean ± SD (µm)	Interval (µm)
Total body length	489,71 ± 30,45	426,84 - 543,98
Width at oesophagus/intestine junction	23,74 ± 2,34	17,53 - 27,70
Length oesophagus	169,47 ± 11,36	124,53 - 183,07
Distance from the excretory pore to the anterior region	90,57 ± 8,86	72,02 - 108,70
Distance from genital primordium to tail	159,71 ± 39,79	76,64 - 223,78
Length genital primordium	32,93 ± 2,63	28,31 - 39,08
Distance from anus to tail	39,69 ± 3,19	33,49 - 46,96

SD: standard deviation.

Table II presents the results of the molecular analysis of L3 larvae naturally infecting four specimens of *T. indica*. The results are presented as Ct values obtained for each DNA concentration. Control samples were prepared using standard strains of *A. costaricensis* and *A. cantonensis*. The results demonstrated that all samples obtained from *T. indica* in qPCR were positive for *A. cantonensis*.

**TABLE II:** Real-time polymerase chain reaction (qPCR) for *Angiostrongylus cantonensis* using the AcantITS1 molecular set

DNA samples	CT	*A. cantonensis*
Negative control-water	No CT	negative
*A. costaricensis* (standard strain)	No CT	negative
*A. cantonensis* (standard strain)	31.93	positive
L3 larvae isolated from the 1st *Tanychlamys indica*	21.01	positive
L3 larvae isolated from the 2nd *T. indica*	20.90	positive
L3 larvae isolated from the 3rd *T. indica*	20.32	positive
L3 larvae isolated from the 4th *T. indica*	20.13	positive

CT: cycle threshold value.

## DISCUSSION

*Tanychlamys indica* is a species of land snail belonging to the family Ariophantidae. The species is native to the Indian subcontinent, including India, Sri Lanka, and parts of Nepal and Bangladesh. It inhabits moist and shaded areas, such as forests, gardens, and agricultural fields.^39^ The accidental introduction of *T. indica* to other regions of the world has occurred as a consequence of human activities, including the trade in plants and agricultural products. The species’ adaptability and rapid reproduction have facilitated its expansion into new areas, particularly in tropical and subtropical regions, where it has found favourable conditions for survival and proliferation.^32^

In addition to the Indian subcontinent, *T. indica* has been documented in a number of other Asian countries, including Saudi Arabia, the Philippines, Indonesia, Malaysia, Japan and Thailand.^27^ ^-^ ^31^ ^,^ ^39^ In these regions, the species has successfully established itself in habitats that are similar to those of its native range. While less prevalent, there are also documented instances of *T. indica* in select regions of South America, the Caribbean, and the United States. In Brazil, the introduction of *T. indica* is associated with the trade in citrus fruit seedlings, initially in the State of Santa Catarina and subsequently in the State of Paraná.^37^

The molluscan specimens were identified as belonging to the species *T. indica*, which represents the inaugural record of this gastropod in the municipality of São Paulo, specifically in the Vila Leopoldina neighbourhood. The area in question covers 7.2 km², has a population of 49.020 inhabitants, a density of 41.93 inhabitants/ha, and a very high Human Development Index (HDI) of 0.907. The district is an important economic hub in the city of São Paulo and is home to the Companhia de Entrepostos e Armazéns Gerais de São Paulo (CEAGESP), which is the largest fruit, vegetable, and flower wholesale centre in Latin America.

The discovery of *T. indica* in the garden area of a municipal school and in the vicinity of the tombs of the Cemitério da Lapa indicates that the species may have been introduced into the municipality via the trade of flowers, grass, ornamental plants, and fruit plant seedlings. This hypothesis is corroborated by the proximity of these businesses to the school and the Cemitério da Lapa, as well as their location just 2 km away from CEAGESP. The larvae of *A. cantonensis* that were recovered from *T. indica* were visually classified as belonging to the larval stage L3 based on their morphometry and morphology. While the tail ending in a fine tip (lanceolate tail) is a typical feature of the species, it is not a precise taxonomic identification factor. However, the L3 measurements were compatible with those obtained by Ash^41^ Thiengo et al*.* ^20^ and Mota et al.^19^

Molecular analysis confirmed the presence of *A. cantonensis* larvae in a population of *T. indica* in an urban area of the city of São Paulo, with an infection rate of 20.0%. In Brazil, the distribution of *A. cantonensis* is closely associated with the dispersal of the African snail *Achatina fulica* Bowdich, 1822, which is considered one of the main potential transmitters of human neural angiostrongyliasis. This is due to the snail’s wide distribution, high population densities and proximity to humans. It is among the top 100 most invasive species globally and represents a significant economic burden as an agricultural pest. Furthermore, it is acknowledged as an urban pest, with documented occurrences in all Brazilian states.^15^ ^-^ ^25^ ^,^ ^45^ The variation in the rate of infection by *A. cantonensis* in different populations of *A. fulica* has been considerable in other studies conducted in Brazil. The prevalence was found to be 21.7% in nine municipalities within the Baixada Santista region^46^ in two other municipalities in Rio de Janeiro, the prevalence was 35.4% in São Gonçalo,^17^ and 10.3% in Barra do Piraí.^17^ In Joinville, SC, the prevalence was 27.4%.^17^ In São Bernardo do Campo/SP, the prevalence was 75%.^47^

At present, over 140 species of mollusc have been identified as natural or experimental intermediate hosts for *A. cantonensis*.^48^ This suggests that *A. cantonensis* has a wide range of intermediate hosts.

Natural infection by *A. cantonensis* in *T. indica* had not been recorded since its introduction into the Southern region of Brazil. This is the first report of *A. cantonensis* infection of *T. indica* in Brazil, thus extending the list of molluscs considered to be intermediate hosts of this nematode in the country to nine species.^11^ ^,^ ^15^ ^-^ ^20^ ^,^ ^22^ ^,^ ^23^ However, *T. indica* had already been reported as an intermediate host of *A. cantonensis* in Bombay, India, together with the slug *Laevicaulis alte* (Férrusac, 1822).^49^

In light of the data presented here, it is imperative to implement a surveillance programme for land molluscs, with particular focus on *T. indica*, in order to gain insight into epidemiological patterns and to facilitate the control of diseases transmitted by this gastropod. Furthermore, this study highlights the necessity for social programmes aimed at raising awareness among local human populations, with the objective of reducing the incidence of human infection, given that cases of neuroangiostrongyliasis have already been documented in the city of São Paulo and in other regions of the country.^15^ ^,^ ^50^ ^,^ ^51^ ^,^ ^52^

It is noteworthy that the area in which *T. indica* was identified as naturally infected is in close proximity (approximately 2 km) to CEAGESP, which oversees the largest public network of warehouses, silos, and granaries in the State of São Paulo, comprising 12 active units distributed across the state. Additionally, the company maintains a network of warehouses (deposits or sales of goods) comprising 13 active units, which are also distributed throughout the State of São Paulo. Among these warehouses is the Entreposto Terminal São Paulo (ETSP), which is the largest supply centre for fruit, vegetables, flowers, fish, and miscellaneous products (garlic, potatoes, onions, dried coconuts, and eggs) in South America. ETSP, where approximately 48.000 individuals and 14.000 vehicles traverse the site on a daily basis,^53^ a scenario that may facilitate the unintentional dispersal of *T. indica* infected with *A. cantonensis* to other regions of the State of São Paulo and the country through the land transportation of fruit, vegetables, flowers and ornamental plants.^2^ ^,^ ^3^

In conclusion, *T. indica* is a species with a complex history of global dispersal, which has been influenced by both human activity and the species’ own adaptive capacity. The advent of this species in new regions presents challenges for both agriculture and public health, underscoring the necessity for efficacious management and control strategies to mitigate its impacts.

It is evident that further research is imperative to map the distribution of *T. indica* in Brazil and to ascertain its potential as an intermediate host of the nematode *A. cantonensis*, as well as to evaluate the economic risks it poses to agriculture. Over the past two decades, there have been reports of human cases of neuroangiostrongyliasis in Brazil, specifically in the states of Rio de Janeiro, Espírito Santo, São Paulo, Minas Gerais, Amazonas, Amapá, Pará, Paraná, Rio Grande do Sul, and Santa Catarina. Furthermore, cases of natural infection of *A. cantonensis* have been documented in molluscs and rodents in various regions of the country.
